# Pharmacological activation of the C5a receptor leads to stimulation of the β-adrenergic receptor and alleviates cognitive impairment in a murine model of familial Alzheimer’s disease

**DOI:** 10.3389/fimmu.2022.947071

**Published:** 2022-08-26

**Authors:** Eleni Fella, Revekka Papacharalambous, Demos Kynigopoulos, Maria Ioannou, Rita Derua, Christiana Christodoulou, Myrto Stylianou, Christos Karaiskos, Alexia Kagiava, Gerasimou Petroula, Chryso Pierides, Maria Kyriakou, Laura Koumas, Paul Costeas, Elena Panayiotou

**Affiliations:** ^1^ Neuropathology Department, Cyprus Institute of Neurology and Genetics, Nicosia, Cyprus; ^2^ Neuromuscular Disorders Center, Cyprus Institute of Neurology and Genetics, Nicosia, Cyprus; ^3^ Laboratory of Protein Phosphorylation and Proteomics, Katholieke Universiteit Leuven, Leuven, Belgium; ^4^ Neuroepidemiology Department, Cyprus Institute of Neurology and Genetics, Nicosia, Cyprus; ^5^ Bioinformatics Department, The Cyprus Institute of Neurology and Genetics, Nicosia, Cyprus; ^6^ Neuroscience Department, Cyprus Institute of Neurology and Genetics, Nicosia, Cyprus; ^7^ Molecular Haematology-Oncology, The Karaiskakio Foundation, Nicosia, Cyprus; ^8^ The Center for the Study of Haematological Malignancies, Nicosia, Cyprus; ^9^ Cellular Pathology-Immunology, The Karaiskakio Foundation, Nicosia, Cyprus; ^10^ Cyprus Cancer Research Institute, Nicosia, Cyprus

**Keywords:** Alzheimer’s disease, β-adrenergic, β-amyloid, C5a receptor, GABA, EP67

## Abstract

Alzheimer’s disease (AD) is a progressive neurodegenerative disease of the brain causing either familial or sporadic dementia. We have previously administered the modified C5a receptor agonist (EP67) for a short period to a transgenic mouse model of AD (5XFAD) and have observed not only reduction in β-amyloid deposition and gliosis but also improvement in cognitive impairment. Inquiring, however, on the effects of EP67 in an already heavily burdened animal, thus representing a more realistic scenario, we treated 6-month-old 5XFAD mice for a period of 14 weeks. We recorded a significant decrease in both fibrillar and pre-fibrillar β-amyloid as well as remarkable amelioration of cognitive impairment. Following proteomic analysis and pathway association, we postulate that these events are triggered through the upregulation of β-adrenergic and GABAergic signaling. In summary, our results reveal how inflammatory responses can be employed in inducing tangible phenotype improvements even in advanced stages of AD.

## Introduction

Alzheimer’s disease (AD), the most common form of dementia worldwide, is a progressive neurodegenerative disorder characterized by amyloid plaque aggregation, cognitive impairment, and memory loss (1). More than 50 million people are currently living with this incurable disease, which is the fifth leading cause of mortality in the world, while 10 million people are newly diagnosed yearly (2, 3). The symptoms of AD are caused by brain degeneration mainly in the area of the cerebral cortex and hippocampus, brain areas highly associated with memory (4).

The microscopic neuropathological hallmarks of AD are the extracellular accumulation of amyloid plaques (senile plaques) and the intracellular formation of neurofibrillary tangles (NFTs). NFTs are composed of hyperphosphorylated Tau protein while the amyloid plaques are made of amyloid precursor protein (APP)-derived Aβ-peptides (5). Monomer Aβ-peptides are highly toxic and aggregate initially as oligomers while further aggregation results in amyloid plaque formation (6). Regarding the amyloid cascade hypothesis, the central feature of AD is the formation and deposition of amyloid plaques around neurons leading to neuroinflammation, synaptic loss, oxidative stress, neuronal death, and NFT formation (7).

Amyloid plaques consist of not only pre-fibrillar and fibrillar forms of Aβ but also proteins such as apolipoprotein E (ApoE) (8, 9) and complement cascade components, such as complement component 1q (1q), cleaved parts of complement component 3 (C3), and complement component 5 (C5), as well as the terminal factors generating the membrane attack complex (MAC) (9, 10). The complement system, a significant mediator of the immune response, has been highly associated with different stages and progression of AD as well as various other neurodegenerative diseases (10, 11). It has previously been shown that the fibrillar form of Aβ-peptide can initiate complement cascade activation (12); however, the exact purpose of the complement’s activation in neurodegeneration has not been fully elucidated yet as both detrimental and beneficial outcomes have been reported (3, 13, 14).

Complement system activation (irrespective of pathway) results in cleavage of the C5 component into complement component 5a (C5a) and complement component 5b (C5b) (15). The C5a peptide is a potent anaphylatoxin that binds the CD88/C5a receptor (C5aR), a member of the G protein-coupled receptor family expressed in various types of cells (16–19), including the central nervous system (CNS) and leukocytes (20). The C5aR is expressed on the membrane of microglia (19, 20) as well as on the membranes of monocytes, macrophages, and neutrophils (8, 21). While microglia are the brain’s resident phagocytes (22), monocytes, macrophages, and neutrophils are the main phagocytes of the periphery (23). The C5a complement component acts as a chemoattractant peptide that recruits C5aR bearing cells in an area with high levels of C5a. C5aR activation results in the active recruitment of these cells, thus enabling phagocytosis and clearance of an area with increased inflammation (20, 24).

Phagocytic cells have been shown to co-localize with amyloid plaques in AD. Both resident microglia and infiltrating phagocytes from the periphery accumulate in areas with amyloid plaques to eliminate pre-fibrillar and fibrillar Aβ-peptides as well amyloid plaques by phagocytosis (25, 26). Peripheral infiltrating monocytes/macrophages go through the blood–brain barrier (BBB) to enter the brain and assist local microglia in amyloid clearance (27). Invasion of infiltrating phagocytic cells in the CNS is a phenomenon extensively observed in many neurodegenerative diseases due to the disruption of the BBB (28, 29). The exact role of phagocytic cells, both resident and invading, in AD has not been fully described. Although phagocytosis itself, through microglia/macrophages, enables catabolism of amyloid plaques, the resulting excessive inflammation may lead to a range of adverse effects (27).

We have recently shown that activation of microglia and macrophages in 5XFAD, a mouse model of AD, eliminates fibrillar and pre-fibrillar amyloid from the brain of affected animals (14). The same effect was observed in a mouse model with peripheral amyloid polyneuropathy, ATTR amyloidosis type I, where recruitment of phagocytic cells in the vicinity of amyloid plaques was followed by pre-fibrillar and amyloid clearance (30). Phagocytic activation was triggered through intermittent administration of a modified C5a agonist molecule, EP67. EP67 is a decapeptide [Tyr-Ser-Phe-Lys-Asp-Met-Pro-(N-methylLeu)-D-Ala-Arg], synthesized based on the last 10 amino acids of the C5a anaphylatoxin. The N-methylation in the leucine amino acid results in conformational changes enabling the molecule to exclusively bind monocytes/macrophages and microglia but not neutrophils. Elimination of its anaphylactic activity, makes EP67 a potent and favorable C5aR agonist molecule since acute inflammation is efficiently bypassed (19, 31, 32).

The exact role of C5a anaphylatoxin and the significance of extended phagocytic activation in AD progression have not been clear since both have been exposed to cause both positive and negative effects in the development and progression of the disease. We have carried out extensive treatment of older 5XFAD mice with the EP67 C5aR agonist. We have found that EP67 administration decreased fibrillar and pre-fibrillar β-amyloid levels while at the same time significantly increased cognition by approximately 280%. Our data indicate that this tremendous upsurge in cognition results from activating the memory-involved β-adrenergic and γ-aminobutyric acid (GABA)ergic pathways, which could provide a feasible strategy in addressing cognitive impairment in AD.

## Methods

### Mice treatment and tissue harvesting

The 5XFAD AD mouse model [Tg6799 on a B6SJL genetic background: Tg(APPSwFlLon,PSEN1*M146L*L286V)6799Vas/Mmjax] has been used in this study. The current mouse model expresses human APP and PSEN1 (Presenilin-1) transgenes having in total five common AD-linked mutations, three on the APP gene [the Swedish (K670N/M671L), Florida (I716V), and London (V717I) mutations] and two on the PSEN1 gene (the M146L and L286V mutations). The 5XFAD mice exhibit most of the features that are related to the AD pathology, in an early and aggressive pattern, including amyloid accumulation, neuronal loss, and gliosis without forming any neurofibrillary tangles (33). The 5XFAD mice have been cross-bred with wild-type (WT) B6SJL hybrid mice and the hemizygous offspring animals were collected for treatment. The WT mice from the same generation were used as age/sex-matched healthy controls. The animals were kept in regular 12-h light/12-h dark cycles and were given free access to water and food, under specific pathogen-free (SPF) conditions. All animal-involving experiments were carried out following the 86/609/EEC Directive. Female and male mice were kept in separate cages until the age of 6 months when treatment was initiated and mice were separated further based on the treatment. The C5aR agonist EP67 (Thermo scientific: P7501-1) was received in the form of lyophilized peptide and given to mice in their drinking water at a concentration of 20 μg/ml. The EP67 peptide was administered to mice the 1st week of their 6th month of age. Following 1-week treatment, the drug was replaced with drinking water until the next treatment, as shown in **Table 1**. The intermittent treatment was carried out following this pattern for 14 weeks until mice completed their 9th month of age.

**Table 1:** Mice treatment protocol.

	Mouse age															
	6th month				7th month				8th month				9th month			
	Weeks				Weeks				Weeks				Weeks			
Treatment group	1	2	3	4	1	2	3	4	1	2	3	4	1	2	3	4
WT H_2_O (*n* = 9)															S	
WT EP67 (*n* = 9)	E				E				E				E		S	
5XFAD H_2_O (*n* = 8)															S	
5XFAD EP67 (*n* = 9)	E				E				E				E		S	

E = EP67.

S = Sacrifice.

Mice were anesthetized using tribromoethanol (Avertin) through intraperitoneal (IP) injection at a dose of 250 mg/kg, sacrificed, and then exsanguinated using 1× phosphate buffered saline (PBS). Brain tissues were harvested, while the two hemispheres were separated. The one hemisphere was frozen at −80°C to be used for techniques requiring frozen tissue, while the other one was paraffin-embedded after fixation in 4% paraformaldehyde (PFA) to be used for immunofluorescence analysis and Thioflavin-S staining.

### Electrophysiology

Pre-processing and analysis of local field potential (LFP) recordings from the CA1 region of the hippocampus was performed on the MATLAB environment (MATLAB r2021b, The MathWorks Inc., Natick, MA, 2015) using custom-made scripts. The sampling frequency was first down-sampled to 1,000 Hz (from 2,000 Hz), before applying a band-stop filter at 50, 100, 150, and 200 Hz. The 10-min-long recordings were segmented to 2-s-long epochs. The power spectral density (PSD) was estimated by fast Fourier transform (FFT) and Bartlett’s method (Bartlett, 1950) using the 2-s-long epochs and a Hamming window, allowing for a frequency resolution of 0.5 Hz. The mean PSD/power ± standard deviation (SD) was calculated across epochs/time for each frequency band of interest: delta (2–6 Hz), theta (6–12 Hz), gamma (30–100 Hz), and sharp-wave ripple (SWR; 120–200 Hz), as these manifest under Avertin-induced anaesthesia (Liu et al, 2020). The spindle frequency range (8–15 Hz) was also characterized. The mean peak power ± SD as well as the frequency at which the peak power occurred ± standard deviation (SD) were also calculated across epochs for each frequency band. Lastly, single spindle events as well as SWR events were detected as described by Stylianou et al. (2020) (34), in order to describe their amplitude, frequency at which they occurred, and instantaneous frequency.

### Immunofluorescence, thioflavin-S staining, and β-amyloid quantification

Sagittal sections (5 μm) from paraffin-embedded brain hemispheres were prepared using a microtome. The sections on the slides were then deparaffinized following the process of overnight incubation at 55°C and then incubation in xylene (3 × 5 min each). Tissue rehydration was obtained by incubation in different concentrations of ethanol (2 × 100%, 90%, and 70%), for 5 min and blocked in 5% bovine serum albumin (BSA) in 1× PBS at room temperature for 1 h. Overnight incubation of the primary antibody [β-Amyloid (B-4), Santa Cruz: sc-28365] was performed followed by 1-h incubation at room temperature of the secondary antibody [Goat anti-Mouse IgG (H+L) Alexa Fluor Plus 555, Invitrogen: A32727]. Thioflavin-S staining was carried out at the same sections, where 1% Thioflavin-S powder (Sigma: T1892) diluted in water was added on the slide for 5 min. Slides were then washed with 50% ethanol for 5 min, rinsed twice with water, and mounted in cover slips using fluorescence mounting medium (DAKO: S3023). Amyloid plaques stained with Thioflavin-S (represented with green color) and Aβ monomers/oligomers stained with the antibody (represented with red color) in the areas of cortex and hippocampus were captured in 5× magnification using a fluorescence microscope (Zeiss Axionvision software, Carl Zeiss Microimaging, Oberkochen, Germany). The images that covered the areas of hippocampus and cortex were jointed in Photoshop to create a single image, and the positive area was quantified using the ImageJ software. The measured staining is represented as a percentage of the red or green positive surface area. Measurements were obtained from five sections per animal and the average was used.

### Enzyme-linked immunosorbent assay for Aβ40 and Aβ 42 amyloid peptides

ELISA was performed in order to measure the levels of Aβ40 and Aβ42 amyloid peptides. For the ELISA experiment, 100 mg from the frozen brain hemispheres was used. Tissues were homogenized and lysed for protein extraction, as explained in detail in the kit’s manuscript (Aβ40, Invitrogen: KMB3481 and Aβ42, Invitrogen: κμβ3441). Briefly, the tissue was initially homogenized by sonication in guanidine-HCl containing lysis buffer, and the supernatant, which was collected upon centrifugation, was further diluted 10 times with 1× PBS supplemented with protease inhibitors (Roche: 11836170001). The samples were further diluted using the standard diluent buffer and applied on the coated plate at the final dilution of 1:20,000 for Aβ42 and 1:500 for Aβ40 measurement. The standards were prepared by performing serial dilutions as described by the protocol, while absorbance was obtained at 450 nm for standards and samples. Samples were used in duplicates or triplicates.

### ELISA for epinephrine (adrenaline), GABA, and insulin

ELISAs were conducted to measure the levels of epinephrine (Assay Genie: MOEB2509), GABA (Assay Genie: MOFI01269), and insulin (Assay Genie: MOFI00142) in the brain, as described by the kit’s manuscript. Mice brains were sonicated for homogenization in 1× PBS supplemented with protease inhibitors. The samples were further diluted using the dilution buffer in 1:100, 1:25, and 1:150, for epinephrine, GABA, and insulin, respectively. The assay was performed at 37°C, and the absorbance of standards and samples was measured at 450 nm. For each ELISA, samples were applied in duplicate.

### Y-maze spontaneous alternation test

Y-maze is a behavioral test that is used to assess short-term spatial and working memory, which is associated with the activity of the hippocampus and represents the cognition status of animals. It is based on mice’s curiosity to explore an unknown place. The animals are placed in the middle of a Y-shaped maze and allowed to explore the maze for 6 min. Ideally, a healthy mouse chooses an alternative arm to enter rather than the one that previously visited, while the one who develops dementia tends to repeat the same arm entry. Mice pre-trial training is not required for the Y-maze test. The arms that mice select to enter are recorded and the percentage of the spontaneous alternations is calculated based on the following formula: number of correct triads/(total number of arms entered − 2) × 100. A correct triad is considered the one that does not include any repeated arm entry (correct triad: BCA, ABC, CAB, etc.) (35).

### Flow cytometry

For the flow cytometry experiment, female 5XFAD and WT mice were treated with EP67 or H_2_O (four animals per group were used) as described in **Table 1**. Whole brain dissociation was performed, immediately upon brain collection, as described in the protocol of Lelios and Greter (2014) (36). The brain was dissociated by dissection in 0.4 mg/ml of collagenase type IV from Clostridium (Gibco: 17104-019), followed by 45-min incubation at 37°C. Before passing the cell suspension through a 70-μm cell strainer, further homogenization was performed by passing several times the dissected tissues through an 18 × 1½ G needle. One wash was performed with 1× PBS, while the separation of leukocytes was obtained by Percoll density gradient medium. Centrifugation for 30 min at 15,000 × *g* at 4°C resulted in the formation of three layers where the middle one contained the desired leukocytes. At the last steps, the top layer was discarded while the other two layers were filtered and the leukocytes were pelleted after 5 min of centrifugation at 450 × *g* at 4°C. Leukocytes were re-suspended in 1 ml of PBS to be used for flow cytometry staining. A modified protocol by Li et al. (2019) (37) was used for cell staining and flow cytometry results analysis.

Before antibody and isotype control staining, viability dye staining was performed in 50 μl of the cells using the Fixable Viability Dye eFluor™ 780 (Thermo Fisher Scientific: 65-0865-14). The cells were then blocked in 1% goat serum supplemented with 0.5% BSA and 2 mM EDTA diluted in 1× PBS at 4°C. Following 10 min of incubation, 5 μl of each of the antibodies or the isotypes were added and 30 min of incubation at 4°C was carried out. For antibody staining, PE-Cy™7 Rat Anti-CD11b (BD: 552850), BV421 Rat Anti-Mouse CD45 (BD: 563890), and APC Rat Anti-Mouse Ly6G (BD: 560599) were used. As isotype controls, the following were selected: Rat DA (BD: 552849), Rat LOU (BD: 562603), and Rat LEW (553932). The stained samples were then washed with flow buffer (1× PBS, 1% BSA, 0.05% sodium azide) and pelleted with certification at 1,500 rpm for 5 min at room temperature. Cells were finally re-suspended in flow buffer and run at a BD FACSAria III flow sorter. Compensation bead staining (Anti-Rat and Anti-Hamster Ig κ/Negative Control Compensation Particles Set, BD: 552845), heat-killed cells stained with a viability dye, and unstained cells were used as controls. Analysis was performed using the FlowJo software.

### Gene expression assay in brain tissue

Two-step reverse transcription quantitative real-time PCR (RT-qPCR) was carried out to quantify the expression levels of various markers in the brain. Brain sections (10 μm) from the paraffin-embedded hemispheres were used to extract total RNA, as described in the manufacturer’s protocol (RNeasy FFPE Kit, QIAGEN: 73504). RNA concentration was assessed by Nanodrop 2000 and maximum 100 ng/μl was used for cDNA synthesis as indicated by protocol. The SuperScript™II Reverse Transcriptase kit (Invitrogen: 18064–022) was used to synthesize first-strand cDNA according to the manufacturer’s instructions. TaqMan Gene Expression Assays for the genes of interest, containing a pair of unlabeled PCR primers and a TaqMan probe with a FAM™ dye label on the 5’ end and a minor groove binder (MGB) non-fluorescent quencher (NFQ) on the 3’ end, were used.

The probes for TaqMan assays that were used are the following: mouse interleukin 6 (IL-6, Thermo Fisher: Mm00446190_m1) and glial fibrillary acidic protein (GFAP, Thermo Fisher: Mm01253033_m1). The glyceraldehyde-3-phosphate dehydrogenase (GAPDH, Thermo Fisher: Mm99999915_g1) gene was used as an endogenous control in delta Ct normalization of samples. For each brain sample, duplicate reactions were run.

### Liquid chromatography with tandem mass spectrometry for brain proteomics discovery and analysis

For LC-MS/MS, about 40 mg of frozen brain tissue was powderized in liquid nitrogen using a mortar and pestle. Brain powder was then homogenized in lysis buffer [8 M urea and 50 mM Tris (pH 8.5)], supplemented with protease inhibitor cocktail at a ratio of 600 µl of lysis buffer per 40 mg of brain powder. Homogenates were allowed to stand at room temperature for 1 h, before being sonicated with a probe-tip sonicator for one up to three 10-s cycles of 1 s on and 1 s off. Homogenates were centrifuged at room temperature for 15 min at 13,000 × rpm. The supernatant was subjected to reduction, alkylation, and quenching with 10 mM DTT, 25 mM iodoacetamide, and 25 mM DTT, respectively. Twenty microliters of the resulting solution was diluted five times in trypsin digestion buffer, containing 50 mM Tris (pH 8.5), 5% CH_3_CN, 0.01% ProteaseMAX (Promega), and 1 µg of modified trypsin (Pierce). After overnight digestion at 37°C, the samples were brought to 1% TFA and centrifuged for 10 min at 14,000 rpm. The supernatant was subjected to desalting with C18 Micro Spin tips (Harvard Apparatus), and 1/60 of the sample was loaded on an Ultimate 3000 UPLC system (Dionex, Thermo Scientific) equipped with an Acclaim PepMap100 pre-column (C18, particle size 3 μm, pore size 100 Å, diameter 75 μm, length 20 mm, Thermo Scientific) and a C18 PepMap analytical column (particle size 2 μm, pore size 100 Å, diameter 50 μm, length 500 mm, Thermo Scientific) using a 4-h linear gradient (300 nl/min) coupled to a Q Exactive Orbitrap mass spectrometer (Thermo Scientific) operated in data-dependent acquisition mode.

Peptides were identified by Mascot (Matrix Science) using UniProt *Mus musculus* concatenated with the CRAPome contaminant database (115,142 sequences) as a database. Carbamidomethylation (C) was included as a fixed modification and oxidation was included (M) as a variable modification. Two missed cleavages were allowed, and peptide tolerance was set at 10 ppm and 20 mmu for MS and MS/MS, respectively. Progenesis software (Nonlinear Dynamics) was used for relative quantification of proteins using the Proteome Discoverer 2.2 Percolator node for peptide validation (FDR < 1%).

Pathway enrichment analysis was performed using the Metascape tool (https://Metascape.org/). Furthermore, terms with a *p*-value of <0.05, a minimum count of 3, and an enrichment factor of >1.5 (the ratio between the observed count and the counts expected by chance) are grouped into clusters based on their membership similarity. *p*-values are calculated based on the accumulative hypergeometric distribution and the *q*-values are based on the Benjamini–Hochberg method for multiple testing. The Kappa scores are used for the similarity metrics to perform hierarchical clustering on the enriched terms obtained, and the subtrees with a similarity of >0.3 are clustered together; only the most statistically significant terms are chosen to be represented within the cluster (38).

Metascape was used to perform enrichment analysis on the untargeted proteomics data obtained for 5XFAD H2O vs. 5XFAD peptide; the Protein Accession ID from UniProt (39) (https://www.uniprot.org/) was used to obtain the gene symbol for each protein. The gene symbol was used as input into Metascape and the organism selected was *Mus musculus*. The KEGG and Gene Ontology (GO) Biological Processes libraries were selected for enrichment analysis; for the identification of biological pathways and processes, the *p*-value cutoff was set to <0.05.

The Reactome online database tool (http://reactome.org) was also used in parallel to Metascape analysis as a means of visualization and data interpretation but was not extensively used as human datasets are mainly used (40, 41).

### Statistical analysis

Statistical analysis was performed using GraphPad Prism version 8.00 for Windows (GraphPad software, San Diego, California, USA) where unpaired Student’s *t*-test was carried out. Using this information, graphical charts representing the data were prepared.

## Results

### Extended intermittent EP67 administration in older mice decreases both amyloidosis and cognitive impairment

**Figure 1:**
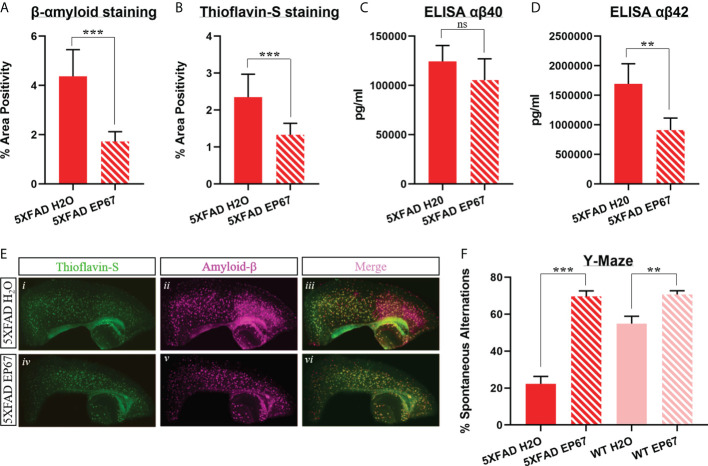
Amyloid load decrease and cognitive impairment amelioration in EP67 treated animals. **(A, B)** Sagittal brain sections from 5XFAD- and 5XFAD EP67-treated mice were co-stained with Thioflavin-S (green; excitation λ: 430; emission λ: 550) and an anti-amyloid-β antibody (red; excitation λ: 555; emission λ: 580). **(A)** Measurements depicting β-amyloid staining were recorded separately, **(B)** as well as measurements representing Thioflavin-S-positive plaques. **(C, D)** Whole hemisphere brain homogenates from 5XFAD and 5XFAD mice treated with EP67 (*via* ELISA) were used to measure the amount of Aβ 40 **(C)** and Aβ 42 **(D)**. **(E)** Representative sections indicating Thioflavin-S and β-amyloid staining in the cortex and hippocampus (composite figures from 200× images). **(F)** 5XFAD- and 5XFAD EP67-treated mice were given the spontaneous alternation behavioral test using a Y-maze. 5XFAD, *n* = 8; 5XFAD EP67,= 9; mean ± SD. **P value ≤ 0.01, ***P value ≤ 0.001; ns, Not Significant.

Starting at 6 months of age, 5XFAD mice were treated in an intermittent manner (as explained in the Methods section) for a period of 14 weeks (**Table 1**). The 5XFAD mice exhibit rapid and severe cerebral amyloidosis as early as 2 months of age. By 6 months of age, this murine model of AD exhibits amyloid plaques throughout the cortex and hippocampus, as well as the brainstem, thalamus, and the olfactory bulb (33). We have previously noted marked cognitive impairment from the age of 3 months (14). Following the duration of treatment, behavioral testing *via* the Y-maze spatial recognition memory assessment was carried out on all mice, including 5XFAD treated and untreated mice, as well as their WT counterparts. We have recorded a noteworthy increase in cognitive acuity in the mice treated with EP67 when compared to untreated 5XFAD mice (**Figure 1F**). Interestingly, WT control mice also treated with EP67 for the same duration as the test group were also positively affected (**Figure 1F**). At the end of treatment, extracted brain samples were then used to quantitate the amounts of β-amyloid and amyloid plaques (Thioflavin-S) through immunohistochemistry (**Figure 1E**). In addition, the amounts of Aβ40 and Aβ42 were also quantified *via* ELISA. Our results show that there is a significant reduction in pre-fibrillar amyloid (**Figure 1A**), Thioflavin-S-positive amyloid plaques (**Figure 1B**), and the Aβ42 β-amyloid species (**Figure 1D**) in mice treated with EP67 for 14 weeks. On the other hand, Aβ40 β-amyloid species (**Figure 1C**) were unaffected by treatment as expected since these species are not prevalent in the mouse model used (33).

### Infiltrating peripheral macrophages/monocytes responsible for amyloid catabolism

Having recorded the significant decrease in amyloid and having previously noted an increase in phagocytosis following treatment with EP67 (14), we set out to conclusively uncover whether this event is a result of resident microglia activation or whether infiltrating peripheral macrophages/monocytes are recruited to assist in the catabolism of β-amyloid plaques.

Microglia are the resident macrophages of the brain, and their role in AD has long been a controversial issue of discussion. Both microglia and astrocytes in the brain are significant modulators of β-amyloid clearance, and both are also known to increase the secretion of IL-6 as a response of their actions. IL-6 is a remarkable cytokine that can have both a pro-inflammatory and an anti-inflammatory effect, depending on the site and conditions of expression (42). In the periphery, macrophages are also known to give rise to the increased expression of IL-6 following phagocytosis (43).

**Figure 2:**
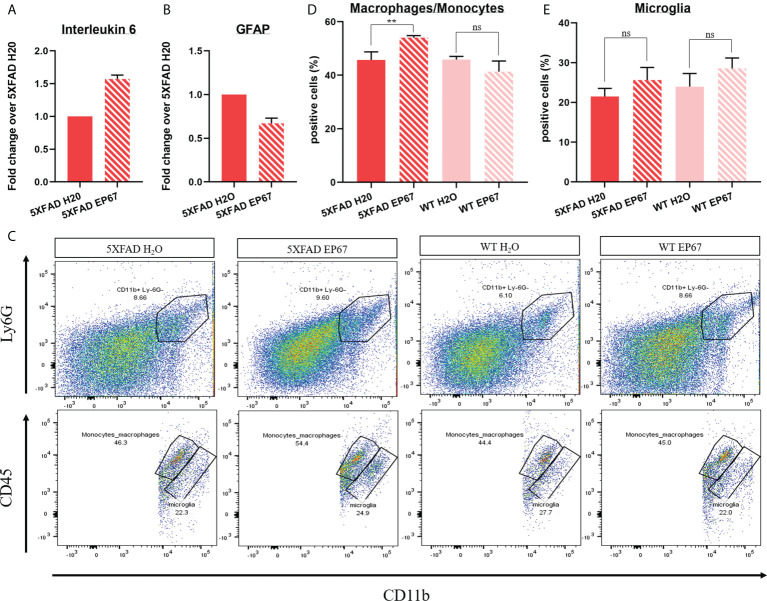
Peripheral infiltrating macrophages assist in amyloid catabolism. **(A, B)** RT-qPCR in 5XFAD- and 5XFAD EP67-treated animals (*n* = 3 both groups, mean ± SEM); transcript levels represent average fold change of treated animals over their controls. Interleukin-6 expression was found to be increased **(A)**, while GFAP expression was decreased **(B)**. **(C–E)** Representative gating exemplifying separation between peripheral macrophages/monocytes (CD45^high^/CD11b^+^) and resident microglia (C45^int^/CD11b^+^) **(C)** and respective quantification indicating an increased amount of macrophages/microglia **(D)** in the brain and an unchanged amount of resident microglia **(E)**. *n* = 4 both groups, mean ± SD. **P value ≤ 0.01; ns, Not Significant.

Following treatment of the 5XFAD mice with EP67, we have noted a significant increase in IL-6 (**Figure 2A**) but, at the same time, a significant decrease in the expression of the astrocyte marker GFAP (**Figure 2B**), indicating little involvement of astrocytes. In order to distinguish, if possible, whether resident phagocytes or infiltrating macrophages are thus involved with β-amyloid clearance, we have chosen a flow cytometry strategy (**Supplementary Figure 1**) that allowed us to isolate and count the CD45^high^/CD11b^+^ population (monocytes/macrophages) versus the C45^int^/CD11b^+^ population (resident microglia) (**Figure 2C**). Our data indicate a significant increase in the infiltrating monocyte/macrophage population (**Figure 2D**) but an unchanged microglia population (**Figure 2E**).

### EP67 treatment triggers GABAergic and β-adrenergic signaling

**Figure 3:**
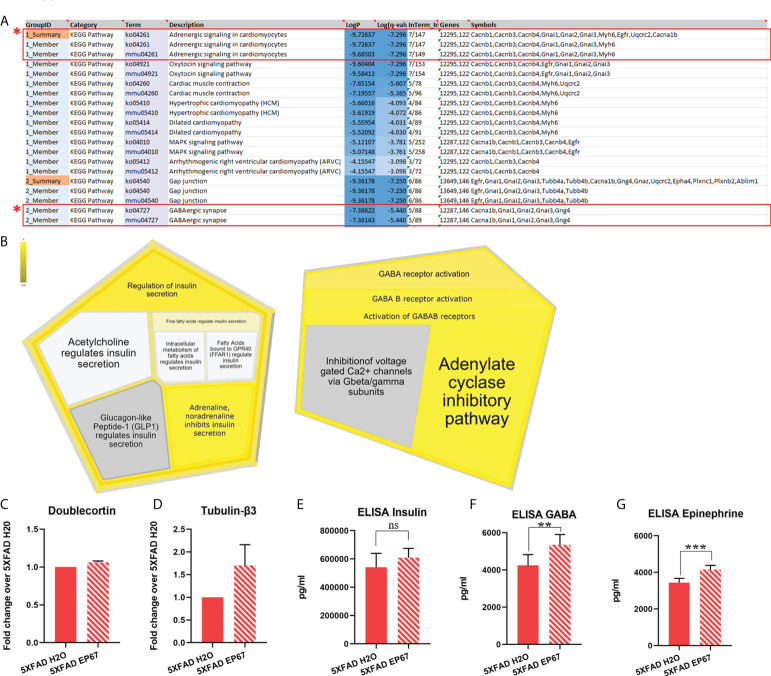
Proteomic analysis implicating β-adrenergic and GABAergic signaling on AD-related cognitive impairment. **(A, B)** Pathway enrichment analysis identifying two major signaling pathways involved with memory consolidation (highlighted), using the Metascape tool **(A)** and the Reactome online database (5XFAD, *n* = 6; 5XFAD EP67, *n* = 6) **(B)**. **(C, D)** RT-qPCR in 5XFAD- and 5XFAD EP67-treated animals (*n* = 3 both groups); transcript levels represent average fold change of treated animals over their controls (*n* = 3, mean ± SEM). **(C)** Unchanged levels of the nascent neuronal cell marker, doublecortin, and non-significant increase in the neuron specific Tubulin-β3 **(D)**. **(E–G)** Whole hemisphere brain homogenates from 5XFAD and 5XFAD mice treated with EP67 (*via* ELISA) were used to measure the amount of insulin **(E)**, GABA **(F)**, and epinephrine **(G)**, which were found to be increased following treatment with EP67 apart from insulin, which remained unchanged. 5XFAD, *n* = 7; 5XFAD EP67, *n* = 9; mean ± SD. **P value ≤ 0.01, ***P value ≤ 0.001; ns, Not Significant.

While the amyloid cascade hypothesis implicates amyloid as the driving force behind the symptomology observed in AD, data suggest that it may not be the driving force behind cognitive impairment since β-amyloid plaques have been found in patients with familial AD at least two decades before any symptoms (44). Our behavioral analysis indicated a significant improvement in cognition; therefore, we carried out RT-qPCR analysis to measure whether the neuronal population was increased following EP67 administration through the developing neuronal marker doublecortin (**Figure 3C**) and the pan-neuronal marker tubulin-β3 (**Figure 3D**). Our results revealed that the neuronal population has remained unchanged throughout treatment.

Therefore, in order to explore whether pathways related to memory consolidation and cognition have been activated through EP67 administration, discovery proteomics were carried out in brain samples of untreated and treated 5XFAD mice. Despite the great number of proteins obtained, we have narrowed down the list to proteins with a *p*-value <0.05 (**Supplementary Table 1**). These proteins were then used for pathway enrichment analysis, which returned a number of results. In terms of involvement in cognition and memory consolidation, we have identified two pathways of interest, adrenergic and GABAergic signaling, using Metascape (**Figure 3A**). Proteins returning a *p*-value <0.05 were used to input for analysis in the online Reactome pathway analysis tool; interestingly, the data again returned pathways related to both the adrenergic and GABAergic receptors (**Figure 3B**). Additionally, we carried out ELISAs and found a significant increase in the amounts of GABA (**Figure 3F**) and epinephrine (adrenaline) (**Figure 3G**), but no differences were detected in the amount of insulin expressed in contrast to the expected pathway from the Reactome database analysis (**Figure 3E**).

### EP67 treatment triggers increased CA1 activity

**Figure 4:**
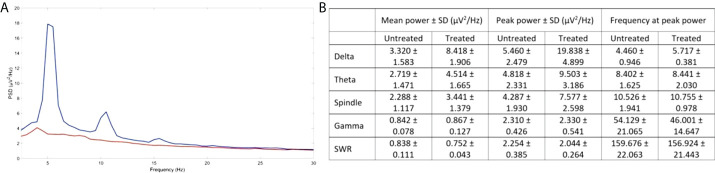
Improved activity in CA1 hippocampal neurons following EP67 treatment. **(A)** PSD of the LFP signal from the CA1 of the hippocampus of one treated (blue) and one untreated animal (orange). **(B)** The mean power spectral density ± SD over epochs, the mean peak power ± SD over epochs and the mean frequency at peak power ± SD over epochs, for the slow oscillation (SO), delta, theta, spindle, gamma, and SWR frequency ranges in the CA1 region of the hippocampus of a treated and an untreated mouse.

Having recorded the significant increase in spatial cognition, as well as the upregulation in β-adrenergic and GABAergic signaling, we have carried out preliminary LFP recordings in the CA1 region of the hippocampus in order to establish whether the observed results would correlate with increased synaptic function. Following recordings from a treated and an untreated 5XFAD animal, our data revealed an increase in spectral power and peak power in sleep-related rhythms, particularly in the slow-oscillation (<2 Hz) and delta (2–6 Hz) frequency ranges in the treated mouse (**Figure 4B**). Of great interest is an increase in peak power in the theta frequency range (6–12 Hz; **Figure 4B**). As this activity did not reflect rapid eye movement (REM) sleep that manifests at this range, we investigated whether it corresponds to hippocampal spindles (45). Indeed, the peak power was also increased in the spindle frequency range (8–15 Hz; **Figure 4**), while we were also able to identify hippocampal spindles with an instantaneous frequency of 10.79 Hz in the treated and 11.04 Hz in the untreated animal. Although the number of spindles does not appear greater in the treated (12 spindles/min) vs. the untreated animal (13 spindles/min), the spindles are of greater amplitude in treated (0.43 μV) vs. untreated (0.33 μV) animals.

## Discussion

A persisting issue with AD is that despite the great number of molecules and compounds developed that have the propensity to reduce amyloid load, there seems to be no positive effect on cognitive impairment, to such an extent that puts the amyloid cascade hypothesis in question (44, 46). Disease-modifying agents for AD must delay or halt the progression of the disease, both in terms of clinical phenotype and in terms of neurodegeneration. For a period of 12 years (starting in 2002), 99% of the agents tested have failed to successfully cross that threshold with only one drug having been approved for further clinical trials during that time frame (47).

We have previously shown that short, intermittent treatment of young 5XFAD mice with a modified C5aR agonist resulted in the reduction of β-amyloid, decrease in gliosis, and marked improvement in spatial working memory (14). Here, we have treated older 5XFAD mice (6 months old), with substantial cerebral amyloidosis, as well as considerable gliosis cognitive impairment in the same intermittent manner for over 3 months. Our results show that treatment of mature 5XFAD mice with EP67 did induce a 60% decrease in Thioflavin-S-positive β-amyloid plaques, as well as in the prominent Aβ42 species (**Figure 1D**), and gliosis, another pathogenic feature of the disease in both mice and humans (**Figure 2B**). We did, however, observe a significant increase in the expression of the pro-inflammatory cytokine IL-6 following EP67 administration (**Figure 2A**). IL-6 is known to be secreted by phagocytes following their interaction with exogenous pathogens and while repairing tissue damage resulting from either trauma or infection (48). Considering that our flow cytometry analysis showed that there are no significant changes in the population of microglia in any of the brain samples examined (with or without EP67 treatment and 5XFAD and WT) but did record a significant increase in the macrophage/monocyte population following treatment with EP67 indicates that enhanced β-amyloid clearance is probably a result of peripheral infiltrating monocytes (**Figures 2C–E**). We have utilized sequential gating strategy that would allow us to successfully distinguish macrophages/monocytes and microglia (CD11b^+^ and Ly6G^-^) but exclude neutrophils and then select C45^int^/CD11b^+^ cells to represent resident microglia and CD45^high^/CD11b^+^ cells representing infiltrating monocytes/macrophages (37). Even though activated microglia in the brain may be considered indistinguishable from peripheral macrophages, due to the damaged BBB, there is an increased infiltration from the periphery. Several lines of evidence suggest that resident microglia take on a pro-inflammatory role, whereas peripheral macrophages appear to instead assist with clearance of the β-amyloid (49), as corroborated here.

As aforementioned, there is considerable criticism surrounding the ability of β-amyloid to promote the cognitive impairment phenotype observed in AD and the ineffectiveness of β-amyloid-reducing compounds to successfully reverse or improve cognitive effects. We thus hypothesized that apart from EP67 affecting β-amyloid clearance, other mechanisms could be affected, ultimately substantially improving spatial memory as observed through behavioral testing. Discovery proteomics followed by pathway-association analysis revealed that pathways involved with cognition become upregulated following EP67 administration. Notably, the β-adrenergic and GABAergic pathways have consistently been over-represented. β-adrenergic receptors in the brain have been associated with the AD cognitive phenotype. These receptors belong to the G-protein-coupled family, responding to mostly the catecholamines epinephrine and norepinephrine and in turn activating the sympathetic nervous system (50). All subtypes of β-adrenergic receptors link to Gs proteins, which are, in turn, linked to adenylate cyclase so that their binding results in an increase in cyclic adenosine monophosphate (cAMP) and eventually protein kinase A (PKA). In AD, Aβ interaction with β_2_-adrenergic receptors results in the internalization and subsequent degradation of the receptors, causing a decrease in adrenergic signaling (51, 52). Furthermore, treatment with the β_2_-adrenergic agonist clenbuterol was shown to improve working memory in aged monkeys and rats (53). Recent epidemiological data, one from a study involving 4 million people and another involving 117 million, show that in terms of neurodegenerative diseases, a deteriorating phenotype in pathophysiology is observed following the use of β-blockers (54, 55).

The association between C5a and adrenergic receptors has previously been investigated, mostly *in vitro*, indicating relationships between the α and β receptors (56, 57). In both cases, while the association is clear, and C5a appears to activate the adrenergic system, it remains uncertain whether the effects are direct or indirect. Monocytes have also been shown to express β-adrenergic receptors, and their activation is considered to be mostly anti-inflammatory (58). Whereas we have not explicitly set out to investigate the association of the modified C5a molecule with adrenergic receptors, we have noticed a specific association of the molecule to the β_2_-adrenergic receptors through the protein prediction database SwissTarget (59) (**Supplementary Figure 2**). As aforementioned, we have also noted a significant upregulation in the proteins associated with GABAergic signaling (**Figure 3B**). GABA is the principal inhibitory neurotransmitter in the brain that appears to be highly involved in the pathology and development of AD. Data from AD patients show diminished GABAergic signaling in both the temporal cortex and the cerebrospinal fluid (CSF), associated with dysfunctional synaptic transmission (60, 61). Furthermore, adrenergic receptors have been shown to regulate GABAergic signaling whereby pharmacological activation of β-adrenergic receptors coupled to Gs increases cAMP levels through adenosine triphosphate (ATP) conversion *via* adenylyl cyclase, thus activating PKA and triggering GABA transporter 1 (GAT-1) phosphorylation, which, in turn, increases the uptake and release of GABA (62). This interaction perhaps describes how an increase of both the adrenergic and GABAergic pathways results in the significant improvement in cognition in the mouse model of AD following treatment with the modified C5aR agonist EP67.

Theta activity in the CA1 region of hippocampus is mostly characteristic of REM activity; however, we have no evidence of REM sleep in these recordings while a strong SO rhythm is evident, which is characteristic of non-REM slow-wave sleep. Hence, we propose that this increased activity in the theta range reflects thalamus-generated spindles that manifest in the cortex and which can also reach the hippocampus *via* the nucleus reuniens or through the entorhinal cortex, both in mice (45) and in humans (63). Cortical spindles directly interact with hippocampal SWRs during non-REM slow-wave sleep and facilitate the process of memory consolidation (64). In fact, increased spindle activity in terms of amplitude and frequency has been directly linked to improved memory consolidation in humans (65, 66), while AD pathology has been associated with a decrease in sleep spindles and in cortical–hippocampal communication during slow-wave sleep (67). Although we have only performed electrophysiological analysis in two animals, we were able to identify some possibly interesting future leads for our research. Hence, we plan to continue this work in more animals using a different anaesthetic, as we postulate that our inability to detect SWRs is likely the result of the suppressive effects of Avertin, as seen, for example, with isoflurane (68).

The role of C5a and its receptors has been shown to be quite contentious in regard to AD pathology. Fonseca et al. have shown that following a 2- to 3-month continuous treatment period with the C5aR antagonist PMX205, a marked reduction in amyloid load and an improvement in cognitive performance were observed (69). In addition, AD mice vaccinated against C5a during the early stage of the disease exhibited reduction in amyloid load, but not those vaccinated during the late stages (70). This result indicates that microglial activation through the C5aR is especially inimical in the early stages of the disease rather than the later stages. On the other hand, complement activation (C3aR) has also been shown to contribute to the clearance of Ab through microglial activation (71). Our results clearly show that following the intermittent treatment of a transgenic mouse model of AD with a modified C5aR agonist not only reduces fibrillar and pre-fibrillar amyloid load in the brain but also enhances spatial memory in a meaningful capacity, thus exerting a significant improvement in the animals’ AD-related pathology. This is a substantial observation in terms of utilizing the body’s inflammatory responses in such a way as to elicit a positive response and addressing cognitive improvement in AD.

## Data availability statement

The datasets presented in this study can be found in online repositories. The names of the repository/repositories and accession number(s) can be found below: http://www.peptideatlas.org/PASS/PASS01761

## Ethics statement

The animal study was reviewed and approved by Cyprus Veterinary Authority.

## Author contributions

Conceptualization and funding: EP. Experimental Design: EF, DK, RD, MS, AK, LK, PC, and EP. Data Collection: EF, RP, DK, MI, RD, CK, AK, PG, CP, and EP. Data Analysis: EF, RD, CC, MS, CP, MK, and EP. Manuscript Preparation: EF, RD, CC, MS, and EP. Manuscript Review: EF, CC, CP, and EP. All authors contributed to the article and approved the submitted version.

## Funding

This work was supported by the Cyprus Research & Innovation Foundation (RIF) under the RESTART program (proposal number: POST-DOC/0718/0113).

## Conflict of interest

The authors declare that the research was conducted in the absence of any commercial or financial relationships that could be construed as a potential conflict of interest.

## Publisher’s note

All claims expressed in this article are solely those of the authors and do not necessarily represent those of their affiliated organizations, or those of the publisher, the editors and the reviewers. Any product that may be evaluated in this article, or claim that may be made by its manufacturer, is not guaranteed or endorsed by the publisher.

## Glossary

**Table:**

Aβ	amyloid-βpeptide
AD	Alzheimer’s disease
APOE	apolipoprotein E
APP	amyloid precursor protein
ATP	adenosine triphosphate
BBB	blood–brain barrier
BSA	bovine serum albumin
C1q	complement component 1q
C3	complement component 3
C5	complement component 5
C5a	complement component 5a
C5b	complement component 5b
C5aR	complement component 5a receptor
cAMP	cyclic adenosine monophosphate
CNS	central nervous system
CSF	cerebrospinal fluid
ELISA	enzyme-linked immunosorbent assay
FFT	fast Fourier transform
GABA	gamma-aminobutyric acid
GAPDH	glyceraldehyde-3-phosphate dehydrogenase
GAT-1	GABA transporter 1
GFAP	glial fibrillary acidic protein
GO	Gene Ontology
IL-6	interleukin 6
IP	intraperitoneal
LC–MS/MS	liquid chromatography with tandem mass spectrometry
LFP	local field potential
MAC	membrane attack complex
MGB	minor groove binder
NFTs	neurofibrillary tangles
NFQ	non-fluorescent quencher
PBS	phosphate buffered saline
PFA	paraformaldehyde
PKA	protein kinase A
PSD	power spectral density
PSEN1	Presenilin-1
REM	rapid eye movement
RT-qPCR	reverse transcription quantitative real-time PCR
SD	standard deviation
SPF	specific pathogen-free
SWR	sharp-wave ripple
WT	wild type
