# Supplementary material for: Pharmacological activation of the C5a receptor leads to stimulation of the β-adrenergic receptor and alleviates cognitive impairment in a murine model of familial Alzheimer’s disease
Source: Front Immunol. 2022 Aug 26;13:947071. doi: 10.3389/fimmu.2022.947071 (PMC9462583; doi:10.3389/fimmu.2022.947071)
Supplement: Supplementary file 1 [file DataSheet_1.pdf]

Supplementary Figures:

Supplementary Fig. 1 Flow cytometry gating strategy. Representative gating of all cells **(a)**, singlets **(b)**, CD11b^{+}Ly-6G^{-} (Q3 population: macrophages/monocytes and microglia but not neutrophils) **(c)** and CD45^{high}/CD11b^{+} (monocytes/macrophages) and C45^{int}/CD11b^{+} (resident microglia) after gating from Q3 population **(d)**.

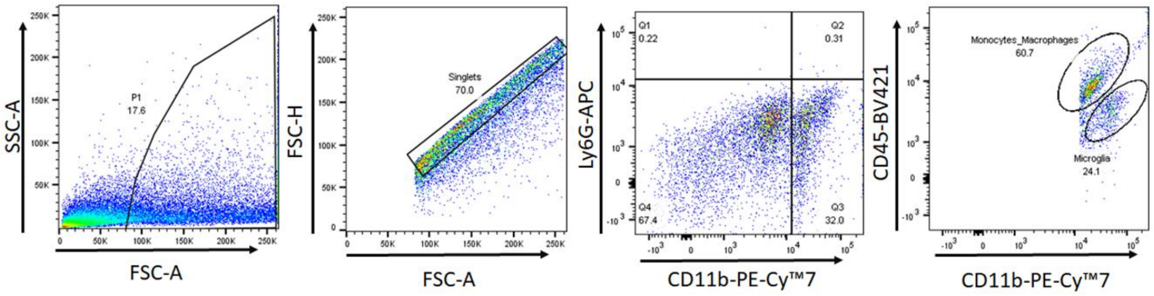

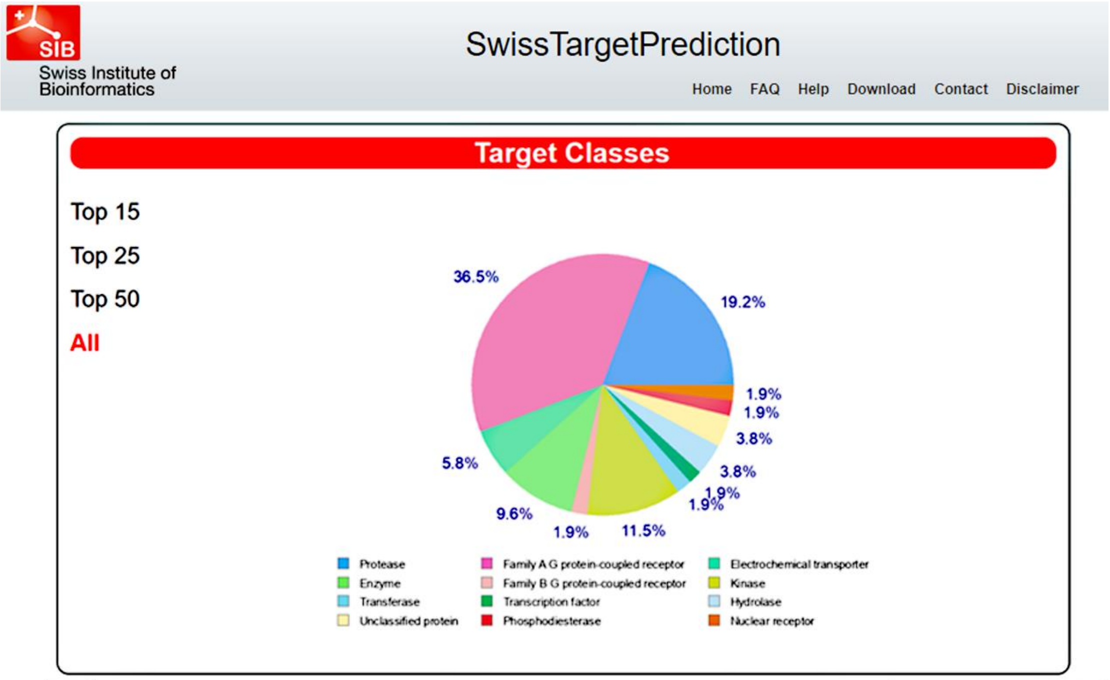

Show 15 entries Search:

Target	Common name	Uniprot ID	ChEMBL ID	Target Class	Probability*	Known actives (3D/2D)
Beta-secretase 1 *(by homology)*	Bace1	P56818	CHEMBL4593	Protease		81 / 126
Endothelin receptor ET-A *(by homology)*	Ednra	Q61614	CHEMBL2286	Family A G protein-coupled receptor		16 / 0
Beta-2 adrenergic receptor *(by homology)*	Adrb2	P18762	CHEMBL3707	Family A G protein-coupled receptor		5 / 0
Epoxide hydratase	Ephx2	P34914	CHEMBL4140	Protease		0 / 13
Dopamine transporter *(by homology)*	Slc6a3	Q61327	CHEMBL2799	Electrochemical transporter		2 / 0
Thrombin *(by homology)*	F2	P19221	CHEMBL1075308	Protease		37 / 95
11-beta-hydroxysteroid dehydrogenase 2 *(by homology)*	Hsd11b2	P51661	CHEMBL3490	Enzyme		1 / 0
Neurokinin 2 receptor *(by homology)*	Tacr2	P30549	CHEMBL2813	Family A G protein-coupled receptor		8 / 0

Supplementary Fig. 2 Swiss Target Prediction.

Supplementary Tables:

Supplementary Table 1. Proteins obtained from the untargeted proteomics analysis with their relevant protein accession ID, gene symbol and p-value.

Protein Accession	Gene Symbol	p-value
E9PV14	Epb41l1	0.004329
A0A0A0MQA3	Serpina1a	0.041125
P15626	Gstm2	0.025974
Q542V3	Srsf4	0.041125
Q3TWW8	Srsf6	0.041125
Q61687	Atrx	0.015152
Q99LB6	Mat2b	0.025974
A0A6I8MX08	Ccdc88a	0.004329
B2KGF0	Rpe	0.025974
P02802	Mt1	0.041125
A0A0R4J0S1	Cdc42ep1	0.025974
Q9WUB3	Pygm	0.041125
Q91WP9	Pygl	0.041125
Q8CJ61	Cmtm4	0.004329
Q3U413	Tsc22d4	0.015152
Q8K2C6	Sirt5	0.015152
B3V097	Cstf2	0.002165
Q8C7E9	Cstf2t	0.002165
Q9CVF5	Nqo2	0.004329
Q3UPE0	Tcea1	0.015152
Q64327	Mea1	0.041125
Q8VDI7	Ubac1	0.041125
Q4V9T8	Eif1	0.0151515
Q9CXU9	Eif1b	0.0151515
Q99KE1	Me2	0.041125
Q8C166	Cpne1	0.015152
Q3TWM2	Gss	0.041125
Q9WVQ5	Apip	0.015152
Q91ZJ5	Ugp2	0.041125
Q69ZX3	Myh11	0.041125
Q5SX39	Myh4	0.041125
Q02566	Myh6	0.041125
Q3UH59	Myh10	0.041125
Q8VDD5	Myh9	0.041125
B1AR69	Myh13	0.041125
K3W4R2	Myh14	0.041125
Q9QYG0	Ndrg2	0.025974
P56213	Gfer	0.041125
A2AER7	Pqbp1	0.025974
Q69ZX3	Myh11	0.00865801
Q8VDD5	Myh9	0.00865801

Q9D0M5	Dynll2	0.041125
P63168	Dynll1	0.041125
Q9D0A3	Arpin	0.025974
G5E8T9	Hagh	0.015152
Q3UJR8	Btf3	0.004329
Q9DC07	Nebl	0.041125
A0A571BG24	Limch1	0.015152
E9QK41	Ablim1	0.008658
P62996	Tra2b	0.041125
Q3UJR8	Btf3	0.041125
Q9D8B3	Chmp4b	0.025974
E9QM73	Wnk2	0.025974
Q3U111	Rdx	0.015152
Q9DCS2	Mettl26	0.008658
Q9JMH6	Txnrd1	0.025974
P85094	Isoc2a	0.025974
***B2RY90**	**Isoc2a**	**0.025974**
Q6PE15	Abhd10	0.025974
Q8C1W9	Nap1l4	0.004329
Q3U0J1	Ppil1	0.025974
Q8BK30	Ndufv3	0.025974
Q8VHL1	Setd7	0.025974
Q8QZS1	Hibch	0.015152
E9PZF0	Gm20390	0.041125
P14824	Anxa6	0.041125
***Q99JX6**	**Anxa6**	**0.041125**
P57759	Erp29	0.041125
P52503	Ndufs6	0.041125
Q64105	Spr	0.041125
Q61206	Pafah1b2	0.015152
Q8BFZ9	Erlin2	0.041125
A0A338P6P7	Pacsin2	0.015152
Q3UYX6	Srsf10	0.041125
P23492	Pnp	0.025974
Q3UDC3	Tom1	0.015152
Q9D6F9	Tubb4a	0.041125
P68372	Tubb4b	0.041125
A5GZX3	Glo1	0.025974
Q9D881	Cox5b-ps	0.041125
P99029	Prdx5	0.041125
Q99LC5	Etfa	0.041125
O35295	Purb	0.041125
Q9DB77	Uqcrc2	0.041125
A0A1W2P832	Ralgapa1	0.025974
A0A087WQ31	Clasp1	0.041125
Q9WVJ2	Psmd13	0.025974
Q542R8	Gnaz	0.008658
Q03137	Epha4	0.041125

***A0A286YDH6**	**Cadps**	**0.015152**
Q80TJ1	Cadps	0.015152
Q3UEJ7	Ass1	0.015152
Q3TYX3	Smyd5	0.041125
E9QN98	Dpp10	0.025974
Q8BKX1	Baiap2	0.041125
O70274	Ptp4a2	0.025974
Q63739	Ptp4a1	0.025974
A0A571BG95	Abr	0.041125
Q3TFU8	Tmed9	0.015152
Q3UG16	Ap1m1	0.041125
P48774	Gstm5	0.015152
Q64519	Sdc3	0.015152
Q3TPX4	Exoc5	0.008658
Q9JI46	Nudt3	0.041125
P0C028	Nudt11	0.041125
Q9DC51	Gnai3	0.025974
P08752	Gnai2	0.025974
B2RSH2	Gnai1	0.025974
A0A1D5RM85	Rpl18a	0.041125
A2ATZ8	Cacnb4	0.041125
P54285	Cacnb3	0.041125
I2FKE4	Cacnb1	0.041125
***A0A0J9YVH8**	**Syngap1**	**0.041125**
F6SEU4	Syngap1	0.041125
P27671	Rasgrf1	0.015152
Q6P9K9	Nrxn3	0.008658
Q9CS84	Nrxn1	0.008658
Q01279	Egfr	0.025974
Q497P1	Stx1a	0.025974
Q8VHY0	Cspg4	0.002165
Q3UT95	Ube2d2a	0.025974
Q3UXZ6	Fam81a	0.025974
Q3UW40	Rpl24	0.025974
Q61037	Tsc2	0.015152
A2AIR7	Cacna1b	0.025974
P50153	Gng4	0.004329
A0A087WS83	Cacna1e	0.025974
P47911	Rpl6	0.015152
B2RXS4	Plxnb2	0.015152
Q9QZC2	Plxnc1	0.015152
Q3UH93	Plxnd1	0.015152
G3X956	Supt16	0.041125
Q9CWK0	Rpl14	0.008658
F7D6J5	Scn8a	0.041125
Q80UT7	Rpl7a	0.015152
Q5KU39	Vps41	0.041125
D3YUQ5	Rundc3b	0.041125

E9Q9B7	Kidins220	0.041125
B2RY09	Etl4	0.004329
Q9WVJ3	Cpq	0.025974

*Duplicated proteins were removed from gene list and were not used for pathway enrichment analysis.
